# Vitamin D deficiency and oral candidiasis in patients with HIV infection: A case‒control study

**DOI:** 10.1186/s12879-024-09065-x

**Published:** 2024-02-19

**Authors:** Shabnam Tehrani, Ladan Abbasian, Seyed Ali Dehghan Manshadi, Malihe Hasannezhad, Sara Ghaderkhani, Amirreza Keyvanfar, Azar Darvishi, AmirHossein Aghdaee

**Affiliations:** 1 Iranian Research Center for HIV/AIDS (IRCHA), Tehran University of Medical Sciences, Tehran, Iran; 2 Infectious Diseases and Tropical Medicine Research Center, Shahid Beheshti University of Medical Sciences, Tehran, Iran; 3 Iranian Research Center for HIV/AIDS (IRCHA), Department of Infectious Diseases, Imam Khomeini Hospital, Tehran University of Medical Sciences, Tehran, Iran; 4 Department of Infectious Disease and Tropical Medicine, School of Medicine, Tehran University of Medical Sciences, Tehran, Iran

**Keywords:** Candidiasis, HIV, Opportunistic infections, Oral candidiasis, Vitamin D

## Abstract

**Background:**

Oral candidiasis is a common opportunistic infection in patients with human immunodeficiency virus (HIV). In addition, most of these patients suffer from vitamin D deficiency. This study aimed to investigate the association between vitamin D levels and oral candidiasis in patients with HIV infection.

**Methods:**

This case‒control study was conducted on HIV-infected patients. Cases were patients with oral candidiasis diagnosed based on physical examinations. Controls were age- and sex-matched individuals without oral candidiasis. The levels of 25-OH vitamin D and other laboratory markers (CD4 count and viral load) were compared between the case and control groups.

**Results:**

A total of 104 cases and 102 controls were included in the study. The cases had significantly lower 25-OH vitamin D_3_ levels (MD = 33.86 ng/mL, 95% CI= (31.85, 35.87), *P* < 0.001) and CD4 counts (MD = 267.48 cells/mm^3^, 95% CI= (189.55, 345.41), *P* < 0.001) than the controls. In addition, viral load was significantly higher in cases than in controls (MD = 7.03 × 10^5^ copies/mL, 95% CI= (4.46 × 10^5^, 9.61 × 10^5^), *P* < 0.001). The multivariate logistic regression analysis revealed that educational status (OR = 0.032, 95% CI= (0.002, 0.100), *P* < 0.001), current HAART (OR = 0.005, 95% CI= (0.001, 0.014), *P* < 0.001), history of oral candidiasis (OR = 20.114, 95% CI= (18.135, 21.957), *P* < 0.001), CD4 count (OR = 0.004, 95% CI= (0.001, 0.006), *P* < 0.001), viral load (OR = 12.181, 95% CI= (1.108, 133.392), *P* < 0.001), and vitamin D level (OR = 0.011, 95% CI= (0.008, 0.015), *P* < 0.001) were significantly associated with the risk of developing oral candidiasis.

**Conclusions:**

Based on the findings, most patients with HIV infection suffer from vitamin D deficiency, especially those with oral candidiasis. Hypovitaminosis D was significantly associated with an increased risk of oral candidiasis. Thus, vitamin D supplementation may assist HIV-positive patients in improving their oral health and preventing oral candidiasis.

## Background

Oral candidiasis is a fungal infection of the tongue and oral cavity that is characterized by the invasion of *Candida* species into mucosal tissues. The majority of the general population carries *Candida spp.* as commensal organisms in the oral cavity. However, they may become pathogenic in immunocompromised hosts and cause opportunistic infections [1]. Predisposing factors for oral candidiasis can be divided into local (e.g., salivary dysfunction, smoking, and denture use) and systemic (e.g., age extremities, malignancies, endocrinopathies, malnutrition, certain medications, and immunocompetent conditions) subcategories [2]. In most cases, the disease manifests as pseudomembranous candidiasis, erythematous candidiasis, and angular cheilitis [3]. The treatment of oral candidiasis is achieved by administering prolonged antifungal medications and confronted with substantial challenges, including drug resistance, biofilm formation, and poor patient compliance [4].

Oral candidiasis is the most common opportunistic infection among patients with HIV infection. Approximately 95% of them experience at least one episode of oral candidiasis throughout their lifetime. It is estimated that over 9 million HIV-positive patients suffer from oral candidiasis worldwide [5]. It may be the initial manifestation of HIV infection or disease progression to acquired immunodeficiency syndrome (AIDS) [6]. Considering the high prevalence of oral candidiasis in HIV-positive patients, studies have focused on modifiable risk factors, such as malnutrition, to prevent it [7].

On the other hand, many individuals with HIV infection suffer from vitamin D deficiency. The prevalence of hypovitaminosis D in HIV-positive patients varies from 21.2% in New York, United States [8] to 92.6% in India [9]. Vitamin D plays a pivotal role in the oral health of patients with HIV by participating in innate and adaptive immune responses. Many immune cells, for instance, T lymphocytes, B lymphocytes, monocytes, and macrophages, express vitamin D receptor (VDR), which initiates intracellular pathways regulating the immune system [10]. HIV-positive patients with hypovitaminosis D are more likely to develop opportunistic infections such as tuberculosis and Cytomegalovirus infections [11]. Nevertheless, the available studies regarding the impact of vitamin D deficiency on oral candidiasis in HIV-infected individuals are limited [12]. Therefore, this study aimed to investigate the association between vitamin D levels and oral candidiasis in HIV-positive patients.

## Methods

### Study design and participants

The present case‒control study was retrospectively conducted at the Imam Khomeini Hospital Complex in Tehran, Iran, from July to October 2023. The study population included HIV-infected patients referred to the HIV clinic of Imam Khomeini Hospital. The inclusion criteria for the cases were as follows: a definitive diagnosis of HIV infection using repeated fourth-generation antigen/antibody combination immunoassay [13, 14], age above 18 years, willingness to participate in the study, and the diagnosis of oral candidiasis during the follow-up time. The control group met all of the above criteria, except for the diagnosis of oral candidiasis. Patients with the following features were excluded from the study: impaired salivary gland function; use of dentures; diabetes mellitus, Cushing’s syndrome, malignancy, and other immunocompetent conditions; and receiving vitamin D supplements, systemic/topical antifungal medications, oral mouthwashes, or corticosteroids in the preceding three months. In this study, samples from the case and control groups were matched by sex and age.

### Sample size and sampling

Samples were recruited based on the consecutive sampling method. Initially, a pilot study was performed, which revealed the 25-OH Vitamin D levels in patients with oral candidiasis (*n* = 10, µ = 20.78, s = 28.45) and patients without oral candidiasis (*n* = 10, µ = 12.45, s = 10.20). Using the following formula, findings of the pilot study, *r* = 1, α = 5%, and power = 80%, the final sample size was estimated to be 206 (103 for each group).

$$ {\text{n}} = \frac{{r + 1}}{r} \times \frac{{\left( {s_1^2 \times s_2^2} \right) \times (A + B)}}{{{d^2}}}$$

### Study procedure and data collection

Eligible samples were interviewed by a trained physician to record baseline characteristics, including age, sex, body mass index (BMI), educational status, and behavioral history. Medical records were reviewed to obtain the medical history and drug history of the patients. The above data were collected in a research-made checklist.

The diagnosis of oral candidiasis was established based on clinical manifestations on physical examinations [15]. Moreover, peripheral venous blood samples were taken from each patient by a trained nurse. They were transferred to the central laboratory of Imam Khomeini Hospital Complex at 20 °C to investigate the CD4 count (cells/mm^3^), viral load (copies/mL), and 25-OH vitamin D level (ng/mL). Vitamin D deficiency was considered as 25-OH vitamin D_3_ < 20 ng/mL [16]. Also, the AIDS was considered as CD4 count < 200 cells/mm^3^ [17].

### Statistical analysis

The data were analyzed using SPSS software version 18.0 (SPSS Inc., Chicago, Illinois, USA). They were described as frequency (%), mean ± standard deviation (SD), mean difference (MD), and 95% confidence interval (CI). Categorical variables were compared between cases and controls using either the chi-square test or Fisher’s exact test, as indicated. Continuous variables were compared between cases and controls using independent-samples *t-*test. Box plots were designed on https://www.statisticskingdom.com. Furthermore, the association between the 25-OH vitamin D level and the risk of developing oral candidiasis was adjusted for confounding variables by applying multivariate logistic regression analysis. Variables with *P* < 0.10 in the univariate analysis were entered into a forward multivariate regression model (Wald method). Finally, findings were reported as odds ratios (ORs) and 95% CIs. In this study, a two-tailed P value below 0.05 was considered statistically significant.

### Ethical considerations

The study was carried out in accordance with the Declaration of Helsinki 2000. The study protocol was approved by the Research Ethics Committees of Imam Khomeini Hospital Complex, Tehran University of Medical Sciences, on June 28, 2023 (IR.TUMS.IKHC.REC.1402.129). Participants completed the written informed consent form.

## Results

### Baseline characteristics, medical history, and physical examinations

Table 1 presents the baseline characteristics and medical history of the patients. A total of 104 cases and 102 controls were included in the study. The baseline characteristics of the samples did not differ between groups, except for educational levels (*P* < 0.001) and drug abuse (*P* < 0.001).The majority of controls and some cases were receiving highly active antiretroviral therapy (HAART) (97.1% vs. 55.8%, *P* < 0.001). Furthermore, the prevalence of previous oral candidiasis was significantly higher in cases than in controls (17.3% vs. 0%, *P* < 0.001). Upon physical examination of patients diagnosed with oral candidiasis (Table 2), pseudomembranous candidiasis (96.2%) was the most common finding, followed by linear gingival erythema (4.8%), xerostomia (1.9%), and acute atrophic candidiasis (1.0%).

**Table 1:** Baseline characteristics and medical history of the patients

Variables	Case (*n* = 104)	Control (*n* = 102)	P value
Baseline characteristics			
Age (years)	43.46 ± 0.9.90	43.45 ± 11.48	0.845^a^
Sex			0.839^b^
Male Female	70(67.3) 34(32.7)	70(68.6) 32(31.4)	
Educational status			< 0.001^b^
Elementary school Secondary school University	71(68.3) 27(26.0) 6(5.7)	39(38.2) 50(49.0) 13(12.8)	
Body mass index (kg/m^2^)	26.56 ± 5.32	27.02 ± 4.53	0.435^a^
Tobacco smoking	53(51.0)	48(47.1)	0.575^b^
Alcohol consumption	15(14.4)	10(9.8)	0.310^b^
Drug abuse	16(15.4)	1(1.0)	< 0.001^c^
Medical history			
Interval from HIV diagnosis (weeks)	20.99 ± 32.59	52.82 ± 46.67	< 0.001^a^
Current HAART	58(55.8)	99(97.1)	< 0.001^b^
Duration of HAART (weeks)	17.45 ± 20.74	53.27 ± 42.23	< 0.001^a^
History of oral candidiasis	18(17.3)	0(0)	< 0.001^a^
Medications in the last 3 months			
Corticosteroids Proton pump inhibitors	2(1.9) 0(0)	0(0) 0(0)	0.498^c^ N/A

Values were described with frequency (%) or mean ± standard deviation

HAART: highly active antiretroviral therapy, HIV: human immunodeficiency virus, N/A: not applicable

^a^ Independent-samples t test, ^b^ Chi-square test, ^c^ Fisher’s exact test

**Table 2:** Clinical manifestations on physical examination of cases (*n* = 104)

Manifestations	Frequency	Percentage
Pseudomembranous candidiasis	100	96.2%
Linear gingival erythema (gingival band)	5	4.8%
Xerostomia	2	1.9%
Acute atrophic candidiasis (erythematous oral patches)	1	1.0%
Chronic atrophic candidiasis (denture stomatitis)	0	0%
Median rhomboid glossitis (central papillary atrophy)	0	0%
Angular cheilitis (perlèche)	0	0%
Hyperplastic candidiasis (mimicker of leukoplakia)	0	0%
Chronic mucocutaneous candidiasis	0	0%
Cheilocandidiasis	0	0%
Chronic multifocal candidiasis	0	0%

### Laboratory findings of the patients

**Fig. 1:**
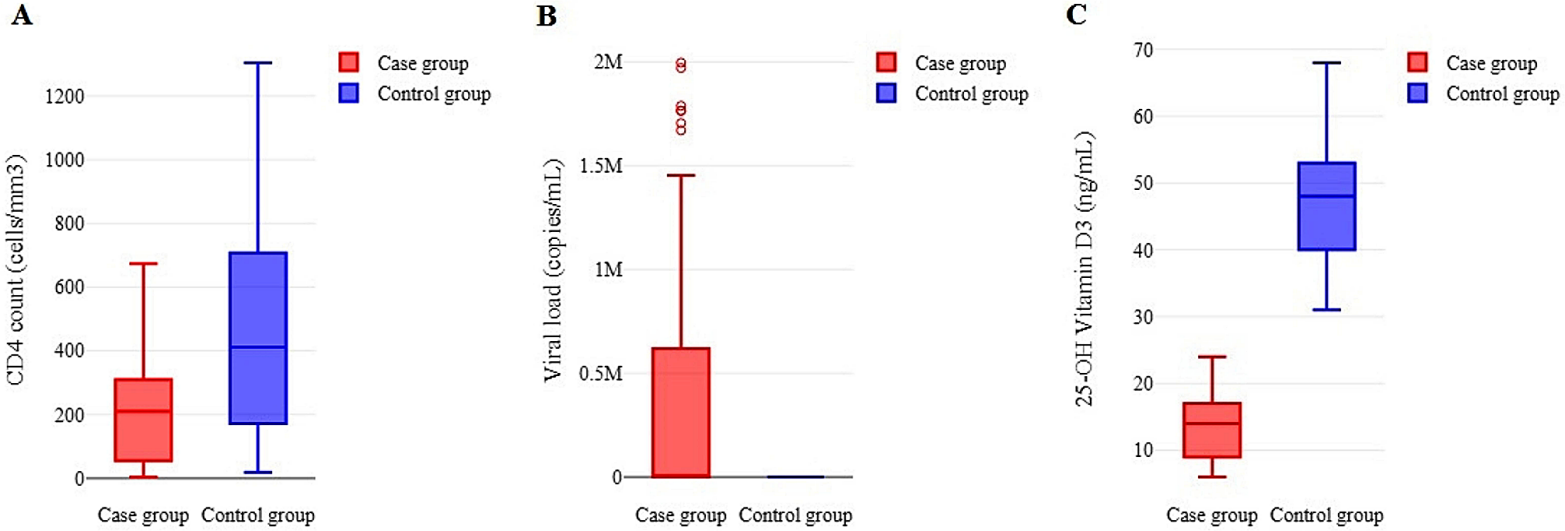
Comparison of laboratory tests between the case and control groups **A**- The cases had significantly lower CD4 counts than the controls (MD = 267.48 cells/mm3, 95% CI= (189.55, 345.41), P < 0.001). **B**- The cases had significantly higher viral loads than the controls (MD = 7.03 × 105 copies/mL, 95% CI= (4.46 × 105, 9.61 × 105), P < 0.001). **C**- The cases had significantly lower 25-OH vitamin D3 levels than the controls (MD = 33.86 ng/mL, 95% CI= (31.85, 35.87), P < 0.001)

**Fig. 2:**
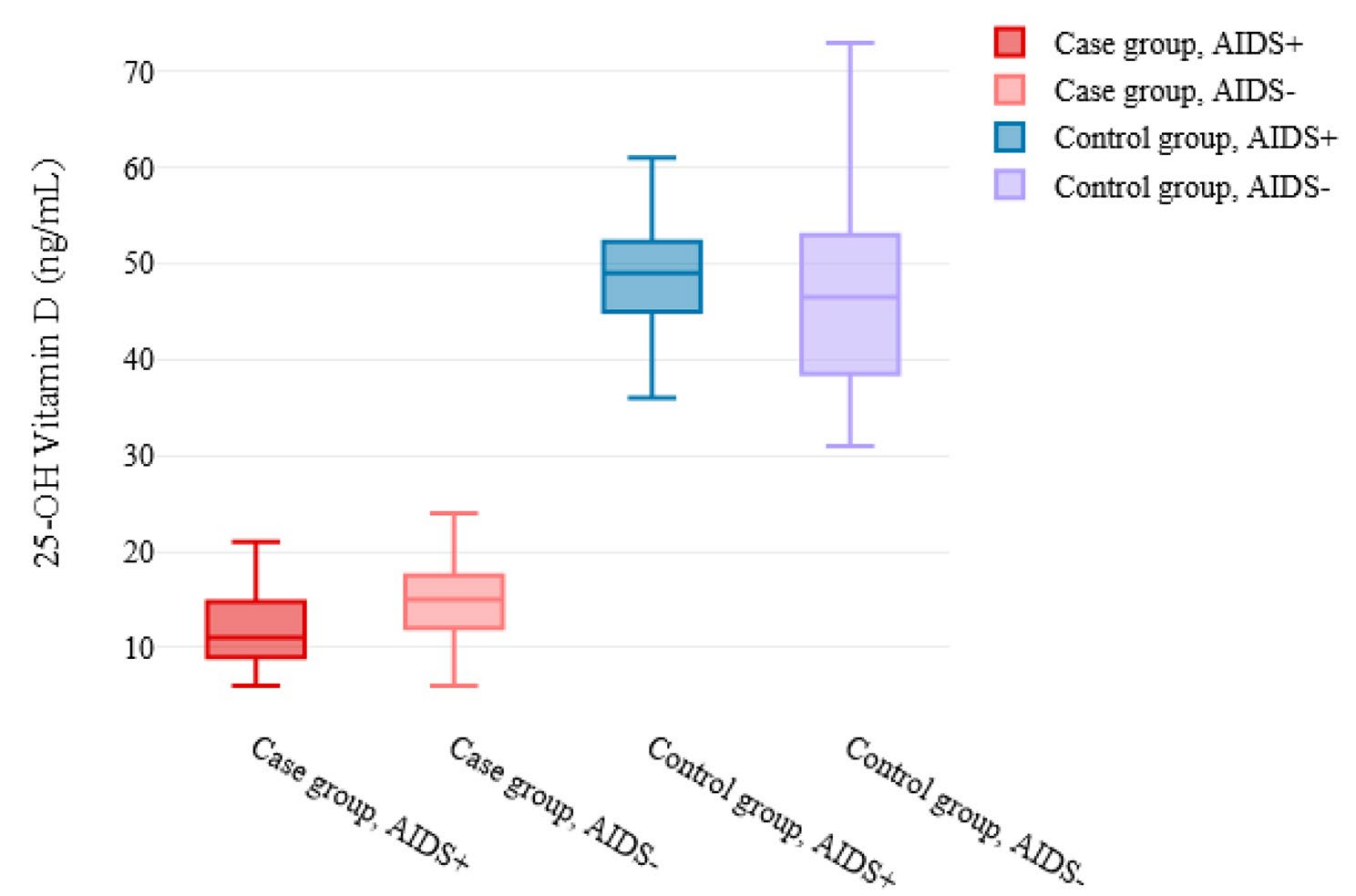
25-OH Vitamin D_3_ levels in the case and control groups by AIDS status

Figure 1 depicts laboratory results of the case and control groups. The cases had significantly lower CD4 counts (208.29 ± 161.16 vs. 475.78 ± 364.63, *P* < 0.001) and 25-OH vitamin D_3_ levels (13.84 ± 4.91 vs. 47.70 ± 9.05, *P* < 0.0001) than the controls. Most patients in the case group had vitamin D deficiency (89.4%). However, none of the patients in the control group had vitamin D deficiency (0%). Further analysis revealed that the prevalence of vitamin D deficiency between the mentioned groups differed significantly (*P* < 0.001). In addition, the viral load was significantly higher in cases than in controls (7.33 × 10^5^±1.31 × 10^6^ vs. 2.96 × 10^4^±1.28 × 10^5^, *P* < 0.001). Figure 2 illustrates 25-OH Vitamin D_3_ levels in the case and control groups by AIDS status. In the case group, patients with AIDS had significantly lower vitamin D_3_ levels than those without AIDS (12.58 ± 5.06 vs. 14.92 ± 4.54, *P* = 0.014). In control group, vitamin D_3_ levels did not differ between those with and without AIDS (48.10 ± 5.06 vs. 47.4 ± 10.41, *P* = 0.703).

### Multivariate logistic regression model

The multivariate analysis revealed that educational status (OR = 0.032, 95% CI= (0.002, 0.100), *P* < 0.001), current HAART (OR = 0.005, 95% CI= (0.001, 0.014), *P* < 0.001), history of oral candidiasis (OR = 20.114, 95% CI= (18.135, 21.957), *P* < 0.001), CD4 count (OR = 0.004, 95% CI= (0.001, 0.006), *P* < 0.001), viral load (OR = 12.181, 95% CI= (1.108, 133.392), *P* < 0.001), and vitamin D level (OR = 0.011, 95% CI= (0.008, 0.015), *P* < 0.001) were significantly associated with the risk of developing oral candidiasis. Table 3 shows findings of the multivariate logistic regression model regarding the effect of vitamin D level on oral candidiasis.

**Table 3:** Logistic regression analysis to adjust the effect of vitamin D level on oral candidiasis

	Unadjusted model		Adjusted model	
	OR (95% CI)	P value	OR (95% CI)	P value
Age	0.983 (0.689, 1.508)	0.852	-	-
Sex		0.667	-	-
Male Female	Reference 0.701 (0.138, 3.546)			
BMI	0.921 (0.679, 1.367)	0.465	-	-
Educational status		0.025		< 0.001
Elementary Secondary and above	Reference 0.067 (0.006, 0.713)		Reference 0.032 (0.002, 0.100)	
Tobacco smoking	0.041 (0.001, 1.723)	0.194	-	-
Alcohol consumption	0.268 (0.068, 1.060)	0.161	-	-
Drug abuse	7.824 (1.862, 32.890)	< 0.001	7.330 (0.075, 720.054)	0.395
Interval from HIV diagnosis	0.921 (0.846, 1.003)	0.158	-	-
Current HAART	0.005 (0.001, 0.010)	< 0.001	0.005 (0.001, 0.014)	< 0.001
History of oral candidiasis	20.589 (19.203, 22.171)	< 0.001	20.114 (18.135, 21.957)	< 0.001
CD4 count (cells/mm3)		0.016		< 0.001
< 200 > 200	Reference 0.120 (0.001, 0.309)		Reference 0.004 (0.001, 0.006)	
Viral load		< 0.001		< 0.001
Undetectable Detectable	Reference 8.000 (5.402, 13.060)		Reference 12.181 (1.108, 133.392)	
25-OH Vitamin D_3_ level	0.521 (0.411, 0.659)	< 0.001	0.011 (0.008, 0.015)	< 0.001

## Discussion

Subsequent to the administration of HAART, the mortality rate of patients with HIV infection dropped dramatically. As the life expectancy of patients is prolonged, they face some chronic complications of HIV infection, such as opportunistic infections [18]. These infections can be prevented and treated by discovering risk factors. Thus, our study was conducted to investigate the effect of vitamin D on oral candidiasis among HIV-positive patients. Based on these findings, hypovitaminosis D was associated with an increased risk of developing oral candidiasis.

In agreement with our findings, Sroussi et al. reported that decreased vitamin D levels and CD4 counts augmented the risk of developing oral candidiasis in HIV-infected patients [12]. It was previously documented that CD4 count correlated with oral manifestations of AIDS [19]. CD4 count and viral load are the primary indicators of disease progression and response to treatment in patients with HIV infection. According to the literature, hypovitaminosis D is associated with decreased CD4 counts and increased viral loads. Vitamin D supplementation may be beneficial to the immune system by elevating CD4 count and diminishing viral load. In other words, vitamin D consumption, when combined with antiretroviral therapy, contributes to viral control [20]. In our study, some of the cases had vitamin D deficiency, even though their CD4 count was over 200 cells/mm^3^. Therefore, it is likely that oral candidiasis will develop in cases of vitamin D deficiency, even with CD4 > 200 cells/mm^3^.

Another study demonstrated that the following factors made HIV-infected individuals more susceptible to oral candidiasis: low CD4 count, high viral load, drug composition or nonuse of HAART, oral carriage of *Candida spp.*, and a history of oral candidiasis [5], which is consistent with our findings. The controls in our study were more than the cases undergoing HAART. Antiretroviral therapy can reduce the risk of developing oral candidiasis through the enhancement of the immune system, secondary to the elevation of CD4 count [21]. A study by Gonçalves et al., in line with our findings, revealed that HIV-infected patients not receiving HAART mostly had oral candidiasis [21]. Interestingly, most patients with HIV infection who are on HAART suffer from hypovitaminosis D, which may be caused by certain antiretroviral drugs, especially Efavirenz. Thus, it is recommended that HIV-positive patients who are getting HAART receive vitamin D supplements [22].

Vitamin D is involved in the regulation of innate and adaptive immunity in combating HIV. The binding of vitamin D to VDR triggers pathways that overexpress anti-HIV molecules. Sufficient vitamin D can induce autophagy in components inhibiting HIV replication [23]. Furthermore, vitamin D plays a crucial role in the local immunity of the oral cavity. It contributes to the expression of antimicrobial peptides (e.g., cathelicidin and β-defensin 2). It participates in autophagy, phagosomal maturation, and antimicrobial activity of macrophages [24]. Hypovitaminosis D makes the oral cavity prone to opportunistic infections, such as oral candidiasis, by interfering with neutrophil recruitment and neutrophil oxidative functions [23]. Hypovitaminosis D can also lead to xerostomia, a condition in which the pathogenic *Candida spp. (*hyphae form) survive rather than the normal commensal flora (budding form). Fungal hyphae adhere to the oral epithelium and invade it by breaking down epithelial integrity. In a confrontation, vitamin D can trigger overexpression of RhoA and Ezrine proteins, which are involved in the enhancement of intercellular connections [25].

The multivariate analysis showed that lower educational status was associated with an increased risk of developing oral candidiasis. Patients with elementary education are less likely to know about health issues. They may have poor oral hygiene, which makes them more susceptible to oral infections. Additionally, a history of oral candidiasis was strongly associated with an increased risk of developing oral candidiasis. This may be due to the colonization of antibiotic-resistant *Candida spp.* in the oral cavity of these patients.

The current study had several limitations. We attempted to manage confounding variables by matching (e.g., age and gender) and restriction (e.g., endocrinopathy, some medications, and others that were excluded from the study). However, there may be some confounders that were not considered. For example, sufficient solar exposure as a natural source of vitamin D could have affected the results. We did not have any data regarding the previous levels of vitamin D over time. It is suggested to follow vitamin D fluctuation in HIV-positive patients over time in a prospective cohort study to discover whether it is associated with oral candidiasis.

## Conclusions

Based on the findings, most patients with HIV infection suffer from vitamin D deficiency, especially those with oral candidiasis. Hypovitaminosis D was significantly associated with an increased risk of oral candidiasis. Thus, vitamin D supplementation may assist HIV-positive patients in improving their oral health and preventing oral candidiasis.

## Data Availability

The datasets used and/or analysed during the current study are available from the corresponding author on reasonable request.
